# Presence and severity of obstructive sleep apnea and remote outcomes of atrial fibrillation ablations — a long-term prospective, cross-sectional cohort study

**DOI:** 10.1007/s11325-014-1102-x

**Published:** 2015-01-08

**Authors:** Filip M. Szymanski, Krzysztof J. Filipiak, Anna E. Platek, Anna Hrynkiewicz-Szymanska, Marcin Kotkowski, Edward Kozluk, Marek Kiliszek, Janusz Sierdzinski, Grzegorz Opolski

**Affiliations:** 1 1st Department of Cardiology, Medical University of Warsaw, 1A Banacha Street, 02-097 Warsaw, Poland; 2 Department of Cardiology, Hypertension and Internal Diseases, Medical University of Warsaw, Warsaw, Poland; 3 Department of Medical Informatics and Telemedicine, Medical University of Warsaw, Warsaw, Poland

**Keywords:** Obstructive sleep apnea, Atrial fibrillation, Sinus rhythm

## Abstract

**Background:**

Prior studies suggested that obstructive sleep apnea (OSA) promotes recurrence of arrhythmia in patients after atrial fibrillation (AF) ablation.

**Methods:**

In this prospective, long-term, observational study, we enrolled 290 consecutive patients admitted for AF ablation. Prior to the ablation, all patients underwent a polygraphy sleep study for the diagnosis of OSA. After the procedure, patients were followed up for mean time of 30 months for AF reoccurrence. OSA was diagnosed when apnea–hypopnea index (AHI) was ≥5. Patients were subsequently divided into groups according to the OSA severity: mild OSA (AHI 5–15/h), moderate OSA (AHI >15 and ≤30/h), and severe (AHI >30/h).

**Results:**

After excluding patients disqualified from the procedure, and those with central sleep apnea, the study population consisted of 251 patients, mean age 57.6 years [163 (64.9 %) male]. OSA was present in 115 (45.8 %) patients, while in 137 (54.6 %) cases, we observed reoccurrence of AF. Recurrence was more often in patients with, than without, OSA (65.2 vs. 45.6 %; *p* = 0.003). We also observed that along with rising OSA severity rose also the number of patients in whom AF was detected during the follow-up period (45.6 vs. 66.2 vs. 57.6 vs. 81.8 %; *p* = 0.005; for non-OSA, mild, moderate, and severe, respectively).

**Conclusions:**

OSA is highly prevalent in AF patients. The presence of OSA lowers chances on successful AF ablation. Early screening, and treatment for OSA in AF patients, may improve low success rates of AF ablation procedures.

## Introduction

Catheter ablation is a definitive method for restoration of sinus rhythm in atrial fibrillation (AF) patients. Unfortunately, the efficacy of the procedure depends on many clinical aspects and varies greatly between patients. Factors influencing ablation outcome include type of AF, and its duration, patient age, comorbidities, and echocardiographic parameters, mostly associated with left atrial diameters [1–5]. Many of these are associated with alteration in intracardiac hemodynamics, causing left atrial overload and its structural and electrical remodeling [6, 7]. One of these is obstructive sleep apnea (OSA), a sleep disorder affecting many patients with AF. OSA contributes negatively to natural history of AF, patients’ risk profile, and prognosis [8–10]. Prior studies have suggested that OSA promotes recurrence of arrhythmia in patients after catheter ablation for AF. A majority of those studies are retrospective or small or include only patients with symptoms of OSA or at high clinical suspicion of the disease. The present study aims to prospectively assess the relationship between OSA and sinus rhythm maintenance in patients after pulmonary vein isolation, in an unselected group of continuous AF patients.

## Materials and methods

### Study population

We designed a prospective, observational cohort study. Consecutive patients with primary diagnosis of AF qualified for first-ever catheter ablation between 2011 and 2013 were enrolled. During a night prior to procedure, all patients underwent a whole-night sleep study for diagnosis of sleep-disordered breathing and were subsequently followed up for a mean of 30 months for arrhythmia recurrence. Only patients without previously diagnosed OSA were included, and inclusion criteria were not based upon any clinical signs or suspicion of underlying sleep-disordered breathing. The study was designed and implemented in accordance with the Declaration of Helsinki, and the study protocol was approved by the University Ethics Committee. Written informed consents for both ablation procedure and study participation were obtained from every patient prior to the enrollment. Declining to sign a consent or its later withdrawal resulted in exclusion from the study.

Inclusion criteria included age ≥18 and ≤75 years and qualification for catheter ablation of AF made prior to the study enrollment, made in accordance with the current guidelines described in detail in the documents of both the European Society of Cardiology and American Heart Association [11, 12]. Paroxysmal AF was defined in accordance with the definitions of the European Society of Cardiology, as self-terminating, usually within 48 h, which may continue for up to 7 days, and persistent AF was defined when AF episode either lasted longer than 7 days or required termination by cardioversion, either with drugs or by direct current cardioversion [11, 13]. Exclusion criteria were as follows: prior ablation for AF; previous diagnosis of OSA; presence of contraindications for polygraphy study including acute and/or chronic pulmonary diseases like obstructive pulmonary disease or active tuberculosis, neuromuscular disease, hemochromatosis, severe neurologic, or psychiatric disorders; diagnosis of central sleep apnea made during the study; contraindications for the ablation procedure including current hyperthyroidism or hypothyroidism or premature termination of the procedure; fatal condition with estimated life expectancy of ≤6 months; or significant cardiac arrhythmia other than AF discovered in electrophysiology during ablation procedure, which forced the operators to switch from pulmonary vein isolation to other types of ablation (i.e., in case of ventricular arrhythmia or atrial flutter).

### Ablation procedure

Radiofrequency catheter ablations were performed by experienced operators in a high-volume, tertiary university hospital, performing approximately 200 AF ablations per year. Ablation procedure was described in detail previously [14]. One quadripolar catheter was placed in the coronary sinus and one in the right ventricle. The left atrium was accessed through a single or double transseptal puncture (or patent foramen ovale, if present) and a 10-pole circumferential 15–25-mm Lasso (Biosense Webster, Diamond Bar, CA, USA) or Optima (St. Jude Medical, Minnetonka, MN, USA), and 4-mm non-irrigated tip ablation (Marinr, Medtronic, Minneapolis, MN, USA) or irrigated Navistar ThermoCool (Biosense Webster, Diamond Bar, CA, USA) catheters were used for mapping and radiofrequency ablation. After transseptal puncture, the patients were heparinized throughout left atrial access (target activated clotting time 300–400 s). Fluoroscopy and an electroanatomic system (CARTO or LocaLisa) were used to navigate the catheters. The following settings were used while delivering radiofrequency energy: non-irrigated tip ablation—temperature limit of 55 °C and a power limit of 35 W; irrigated tip ablation—temperature limit 48 °C, power limit 35 and 30 W on posterior wall. All electrograms were displayed on an electrophysiological recording system. The endpoint of the procedure was the isolation of pulmonary vein potentials (PVP) in all pulmonary veins. If the patient was on AF, cardioversion was performed to verify the isolation during sinus rhythm.

### Diagnosis of OSA

In every patient, an overnight sleep study was performed using a portable device for diagnosing the sleep disorders (Embletta Gold; Flaga, Reykjavik, Iceland), which is a level 3 sleep monitoring tool, according to recommendations of the European Respiratory Society and the European Society of Hypertension feasible for objective confirmation of OSA diagnosis [15]. The device recorded data from greater than four channels including respiratory movements or respiratory effort, airflow, heart rate, ECG, and oxygen saturation. To avoid bias, obtained data were manually scored by an experienced physician specialized in sleep medicine, with respect to the current definitions and recommendations of the American Academy of Sleep Medicine [16]. Apnea was defined as a cessation of airflow lasting 10 s, and hypopnea was defined as a recognizable transient reduction (but not complete cessation) of breathing for ≥10 s, a decrease of greater than 50 % in the amplitude of a validated measure of breathing, or a reduction in amplitude of less than 50 % associated with oxygen desaturation of 4 % or more. Desaturation was defined a blood oxygen saturation of 90 % or lower. Contrary to other studies (which focus rather on OSA syndrome), diagnosis of OSA was made solely when the apnea–hypopnea index (AHI) in the recorded study was 5/h or more, irrespectively to daytime OSA symptoms, which allowed objective evaluation of the disease severity. Patients were subsequently divided into groups according to the OSA severity: Mild OSA was defined as an AHI between 5 and 15, moderate OSA was an AHI of >15 and ≤30/h, and AHI >30/h was the criterion for severe OSA. All patients with diagnosed OSA were managed according to the current guidelines, and those with an AHI of >15/h or AHI >5 with symptoms of excessive daytime sleepiness were offered a continuous positive airway pressure treatment [16]. Moreover, patients with body mass index ≥35 kg/m^2^ were referred to a clinic specializing in treatment of obesity and bariatric surgery.

### Follow-up methods

Primary endpoint of the study was reoccurrence of the AF, defined as at least one AF episode of at any moment during the follow-up period. Screening methods for AF have various algorithms in different clinical trials. We applied criteria extrapolated from those defined in the Heart Rhythm Society/European Heart Rhythm Association/European Cardiac Arrhythmia Society consensus statement, which included AF of at least 30 s’ duration when assessed with ECG monitoring, including 24-h ECG Holter monitoring, implantable pacemakers, implantable defibrillators, and subcutaneous ECG monitoring devices [17]. Time points for ECG Holter screening were chosen according to the clinical status and decision of an attending physician, with at least one mandatory ECG Holter during the first 6 and 12 months after the procedure. We also included an additional screening criterion; if patients reported symptoms (i.e., heart palpitations or syncope), which were electrocardiographically documented to be associated with AF, previously to ablation, we considered recurrence of patients' self-reported symptoms after the procedure (even when not ECG-documented) equal to AF recurrence.

### Statistical analysis

Continuous data are presented as mean ± standard deviation (SD) and were compared using the Mann–Whitney test or Student’s *t* test. Categorical variables were compared using either the chi-square or Fisher exact tests. A *p* value of less than 0.05 was considered statistically significant, whereas the confidence intervals (CI) were 95 %. The analysis of variance was used to compare multiple variables. Multivariate logistic regression analysis was performed to identify the independent risk factors of AF recurrence. Statistical analyses were performed using SAS statistical software version 8.02 (SAS Institute, Inc., Cary, NC, USA).

## Results

Study population consisted of 290 patients; in 22 cases, sleep study was not preformed due to technical issues, lack of patient’s cooperation, or quality of recorded data was insufficient to perform analysis. Of 268 remaining cases, in 14 patients, AF ablation was not performed. The procedure was terminated due to complications or electrophysiological study revealed arrhythmia not suitable for pulmonary vein isolation. In three cases, sleep study revealed central sleep apnea.

Of the remaining 251 patients, at the age of 57.6 ± 10.0 years, 163 (64.9 %) were male. Regarding the cardiovascular risk factors, mean body mass index in the population was 29.6 ± 4.8 kg/m^2^, and hypertension and dyslipidemia were highly prevalent (respectively, in 72.9 and 62.9 % of patients). The baseline characteristics of the study population are given in Table 1.

**Table 1:** Baseline characteristics of the study population

Parameter	Mean ± SD or *n* (%)
Male sex	163 (64.9 %)
Age (years)	57.6 ± 10.0
Height (cm)	172.0 ± 10.3
Weight (kg)	87.6 ± 16.4
Body mass index (kg/m^2^)	29.6 ± 4.8
Neck circumference (cm)	40.2 ± 3.6
Waist circumference (cm)	108.1 ± 66.1
SBP (mm Hg)	131.5 ± 16.5
DBP (mm Hg)	80.6 ± 11.1
HR (beats per min)	74.8 ± 15.5
Prior myocardial infarction	20 (8.0 %)
Diabetes mellitus	21 (8.4 %)
Hypertension	183 (72.9 %)
Gout	17 (6.8 %)
Smoking	28 (11.2 %)
Alcohol consumption	23 (9.2 %)
Prior stroke	21 (8.4 %)
Peptic ulcer disease	44 (17.5 %)
Dyslipidemia	158 (62.9 %)
Family history of CVD	100 (39.8 %)
Paroxysmal AF	176 (70.1 %)

*SD* standard deviation, *SBP* systolic blood pressure, *DBP* diastolic blood pressure, *HR* heart rate, *CVD* cardiovascular disease, *AF* atrial fibrillation

Results of the sleep study showed that OSA (AHI ≥5/h) was present in 114 (45.4 %) patients. Mean AHI was 15.4 ± 12.2, mean lowest registered blood oxygen saturation was 81.8 ± 6.5 %, and total time in desaturation was 26.8 ± 43.1 min.

OSA patients were older (59.7 ± 7.8 vs. 55.7 ± 11.3 kg, *p* = 0.01), had higher body mass index (30.8 ± 5.3 vs. 28.5 ± 4.1 kg/m^2^, *p* = 0.0002), had higher neck circumference (41.2 ± 3.8 vs. 39.3 ± 3.3 cm, *p* = 0.0001), and had higher waist circumference (108.6 ± 12.6 vs. 107.6 ± 88.1 cm, *p* < 0.0001) than patients without OSA. Paroxysmal form of the arrhythmia was more often noticed in non-OSA patients, while the OSA group more often had persistent AF (*p* = 0.05); detailed results are presented in Table 2.

**Table 2:** Comparison of patients with and without obstructive sleep apnea (OSA)

Parameter; mean ± SD or *n* (%)	OSA+ (*n* = 115)	OSA– (*n* = 136)	*p* value
AHI (per hour)	15.4 ± 12.2	1.9 ± 1.4	**–**
Lowest SpO_2_ (%)	81.8 ± 6.5	86.5 ± 5.0	**<0.0001**
Time in desaturation (min)	26.8 ± 43.1	12.3 ± 36.2	**<0.0001**
Percent time in desaturation (%)	5.8 ± 10.0	3.2 ± 9.9	**<0.0001**
Oxygen desaturation events (per hour)	15.9 ± 13.0	3.1 ± 3.3	**<0.0001**
Snoring percent time	12.4 ± 19.7	10.6 ± 18.6	0.11
AF recurrence	75 (65.2 %)	62 (45.6 %)	**0.003**
Male sex; *n* (%)	80 (69.6 %)	83 (61.0 %)	0.20
Age (years)	59.7 ± 7.8	55.7 ± 11.3	**0.01**
Height (cm)	172.0 ± 9.9	172.0 ± 10.7	0.97
Weight (kg)	91.1 ± 17.0	84.6 ± 15.3	**0.002**
Body mass index (kg/m^2^)	30.8 ± 5.3	28.5 ± 4.1	**0.0002**
Neck circumference (cm)	41.2 ± 3.8	39.3 ± 3.3	**0.0001**
Waist circumference (cm)	108.6 ± 12.6	107.6 ± 88.1	**<0.0001**
SBP (mm Hg)	132.9 ± 16.7	130.3 ± 16.3	0.23
DBP (mm Hg)	82.0 ± 11.5	79.4 ± 10.7	**0.06**
HR (beats per min)	76.5 ± 17.4	73.3 ± 13.6	0.13
Prior MI	9 (7.8 %)	11 (8.1 %)	0.87
Diabetes mellitus	12 (10.4 %)	9 (6.6 %)	0.39
Hypertension	91 (79.1 %)	92 (67.6 %)	**0.06**
Gout	11 (9.6 %)	6 (4.4 %)	0.17
Smoking	10 (8.7 %)	18 (13.2 %)	0.35
Alcohol consumption	13 (11.2 %)	10 (7.4 %)	0.39
Prior stroke	13 (11.3 %)	8 (5.9 %)	0.19
Dyslipidemia	74 (64.3 %)	84 (61.8 %)	0.77
Family history of CVD	41 (35.7 %)	59 (43.4 %)	0.39
Paroxysmal AF	73 (63.5 %)	103 (75.7 %)	0.05

Desaturation = blood O_2_ saturation <90 %

*SD* standard deviation, *OSA* obstructive sleep apnea, *Lowest SpO*_*2*_ lowest registered blood oxygen saturation, *SBP* systolic blood pressure, *DBP* diastolic blood pressure, *HR* heart rate, *MI* myocardial infarction, *CVD* cardiovascular disease, *AF* atrial fibrillation

Values in bold indicate statistical significance

Patients were followed up for a mean time of 30 months. We observed the AF recurrence in 137 (54.6 %) patients, while 114 of them remained free from arrhythmia during the whole follow-up period. Recurrence was more often observed in the group with OSA (65.2 vs. 45.6 %; *p* = 0.003) than in the group without this disorder. Except for higher heart rate on admission (77.3 ± 16.3 vs. 71.8 ± 14.1/min, *p* = 0.005), there were no statistical differences between the group in sinus rhythm and patients in whom AF recurrence was observed (Table 3).

**Table 3:** Comparison of patients with sinus rhythm and recurrence of atrial fibrillation in 30 months following ablation procedure

Parameter; mean ± SD or *n* (%)	Sinus rhythm (*n* = 114)	Atrial fibrillation recurrence (*n* = 137)	*p* value
AHI (per hour)	6.2 ± 7.7	9.7 ± 12.5	**0.01**
Lowest SpO_2_ (%)	85.0 ± 5.8	84.1 ± 6.4	0.25
Time in desaturation (min)	13.4 ± 28.4	22.7 ± 47.0	0.11
Percent time in desaturation (%)	3.5 ± 8.7	5.1 ± 11.0	0.16
Oxygen desaturation events (per hour)	6.5 ± 6.9	10.5 ± 13.1	**0.03**
Snoring percent time (%)	11.8 ± 19.6	11.1 ± 18.7	0.78
OSA	40 (35.1 %)	75 (54.7 %)	**0.003**
Male sex	72 (63.2 %)	91 (66.4 %)	0.68
Age (years)	57.6 ± 10.5	57.5 ± 9.6	0.55
Height (cm)	171.4 ± 10.2	172.5 ± 10.4	0.42
Weight (kg)	86.1 ± 14.7	88.8 ± 17.6	0.19
Body mass index (kg/m^2^)	29.3 ± 4.1	29.8 ± 5.4	0.58
Neck circumference (cm)	40.0 ± 3.1	40.3 ± 4.1	0.61
Waist circumference (cm)	111.7 ± 95.3	104.9 ± 13.6	0.53
SBP (mm Hg)	132.3 ± 16.8	130.8 ± 16.4	0.49
DBP (mm Hg)	80.6 ± 10.9	80.6 ± 11.4	0.99
HR (beats per min)	71.8 ± 14.1	77.3 ± 16.3	**0.005**
Prior MI	9 (7.9 %)	11 (8.0 %)	0.85
Diabetes mellitus	9 (7.9 %)	12 (8.8 %)	0.99
Hypertension	79 (69.3 %)	104 (75.9 %)	0.30
Gout	6 (5.3 %)	11 (8.0 %)	0.54
Smoking	13 (11.4 %)	15 (10.9 %)	0.93
Alcohol consumption	9 (7.9 %)	14 (10.2 %)	0.68
Prior stroke	6 (5.3 %)	15 (10.9 %)	0.16
Dyslipidemia	71 (62.3 %)	87 (63.5 %)	0.95
Family history of CVD	43 (37.7 %)	57 (41.6 %)	0.90
Paroxysmal AF	86 (75.4 %)	90 (65.7 %)	0.12

Desaturation = blood O_2_ saturation <90 %

*SD* standard deviation, *OSA* obstructive sleep apnea, *Lowest SpO*_*2*_ lowest registered blood oxygen saturation, *SBP* systolic blood pressure, *DBP* diastolic blood pressure, *HR* heart rate, *MI* myocardial infarction, *CVD* cardiovascular disease, *AF* atrial fibrillation

Values in bold indicate statistical significance

When we divided patients into prespecified groups according to AHI, we observed that along with rising severity of OSA rose also the number of patients in whom AF was detected during the follow-up period (45.6 vs. 66.2 vs. 57.6 vs. 81.8 %; *p* for trend = 0.005; for non-OSA and mild, moderate, and severe OSA, respectively). Only other significant differences between the groups with different AHI values concerned only polygraphy parameters (Table 4).

**Table 4:** Differences between the groups according to obstructive sleep apnea (OSA) severity

Parameter; mean ± SD or *n* (%)	Mild OSA (*n* = 71)	Moderate OSA (*n* = 33)	Severe OSA (*n* = 11)
AHI (per hour)	8.6 ± 2.5	20.9 ± 3.7	43.4 ± 15.8
Time in desaturation (min)	20.4 ± 32.4**	22.5 ± 28.6***	105.8 ± 87.0**, ***
Lowest SpO_2_ (%)	83.1 ± 5.4**	81.6 ± 5.5***	65.9 ± 11.4**, ***
Percent time in desaturation (%)	5.1 ± 8.5	4.3 ± 6.7	13.9 ± 20.1
Oxygen desaturation events (per hour)	10.4 ± 5.0*, **	21.7 ± 5.7*, ***	47.9 ± 26.2**, ***
Snoring percent time	11.1 ± 18.7	13.3 ± 20.5	17.9 ± 23.9
Male sex	52 (73.2 %)	23 (69.7 %)	5 (45.5 %)
Age (years)	59.6 ± 8.5	60.0 ± 7.2	58.6 ± 7.0
Height (cm)	172.0 ± 9.0	173.2 ± 11.6	169.8 ± 11.3
Weight (kg)	89.2 ± 18.1	93.8 ± 15.1	99.4 ± 22.0
Body mass index (kg/m^2^)	30.1 ± 5.5	31.3 ± 4.6	34.4 ± 6.3
Neck circumference (cm)	41.1 ± 3.9	41.1 ± 3.4	42.0 ± 4.5
Waist circumference (cm)	106.9 ± 13.2	109.4 ± 11.2	116.9 ± 15.8
SBP (mm Hg)	133.2 ± 16.6	132.1 ± 18.3	131.7 ± 15.1
DBP (mm Hg)	82.8 ± 10.7	81.0 ± 12.9	79.6 ± 12.1
HR (beats per min)	75.4 ± 18.7	80.7 ± 16.7	79.6 ± 15.8
Prior MI	6 (8.5 %)	1 (3.0 %)	2 (18.2 %)
Diabetes mellitus	4 (5.6 %)	6 (18.2 %)	2 (18.2 %)
Hypertension	54 (76.1 %)	28 (84.8 %)	9 (81.8 %)
Gout	8 (11.3 %)	1 (3.0 %)	2 (18.2 %)
Smoking	6 (8.5 %)	3 (9.1 %)	1 (9.1 %)
Alcohol consumption	6 (8.5 %)	6 (18.2 %)	1 (9.1 %)
Prior stroke	9 (12.7 %)	3 (9.1 %)	1 (9.1 %)
Peptic ulcer disease	15 (21.1 %)	5 (15.2 %)	2 (18.2 %)
Dyslipidemia	44 (62.0 %)	22 (66.7 %)	8 (72.7 %)
Family history of CVD	23 (32.4 %)	14 (42.4 %)	4 (36.4 %)

*SD* standard deviation, *OSA* obstructive sleep apnea, *Lowest SpO*_*2*_ lowest registered blood oxygen saturation, *Total desaturation time* time of blood oxygen saturation <90 %, *SBP* systolic blood pressure, *DBP* diastolic blood pressure, *HR* heart rate, *MI* myocardial infarction, *CVD* cardiovascular disease, *AF* atrial fibrillation

Statistical significance (*p* < 0.05): *significant difference between mild and moderate OSA group; **significant difference between mild and severe OSA group; ***Significant difference between moderate and severe OSA group

In the multiple logistic regression analysis, OSA was the strongest, independent risk factor for AF recurrence (odds ratio [OR] 2.58; 95 % CI 1.52–4.38; *p* < 0.0001); another risk factor was history of hyperthyroidism (OR 2.21; 95 % CI 1.91–4.10; *p* = 0.01) (Table 5).

**Table 5:** Independent risk factor for AF recurrence

	Odds ratio	95 % CI	*p* value
Obstructive sleep apnea	2.58	1.52–4.38	<0.0001
History of hyperthyroidism	2.21	1.91–4.10	0.012

*CI* confidence interval

## Discussion

The relationship between AF and OSA is very complex. Many studies have been conducted in order to establish how OSA promotes the arrhythmia, what is the prevalence of OSA in AF patients, and if treatment strategies should depend on OSA and AF coexistence [18–20]. OSA is one of the major problems in AF patients, contributing negatively not only to disease course but also to increasing the stroke risk [21]. Its prevalence is shown to be over 40 % [22]. Patients qualified for AF ablation tend to be different, for example, in terms of age and comorbidities than the general AF population, but currently, a lot of bias can be encountered in qualification for AF [23]. We have previously reported that in patients qualified for AF ablation, OSA prevalence is over 45 %, as it is in the case of this study [19]. In relationship to the general population, OSA rates in AF patients are much higher. In the general population, OSA is estimated to affect 24 % of men and 9 % of women, at the age of 30 to 60 years [24].

Causes of this often coexistence are associated with sleep apnea pathophysiology. Intermittent hypoxia and hypercapnia caused by apnea are associated with sympathetic and chemoreflex activation, which lead to vasoconstriction and increase blood pressure [25, 26]. The presence of OSA affects also arterial stiffness, causes endothelial dysfunction and promotes atherogenesis and left atrial enlargement, and leads to aberrant conduction and as a consequence AF [27, 28]. OSA was also shown to affect cardiac electrical stability, increase transmural pressure gradient, and distort cardiac configuration [29]. All those mechanisms were shown to promote de novo occurrence of AF but are responsible in an equal manner for recurrence of the arrhythmia after invasive treatment.

In AF patients who undergo catheter ablation procedures, defining procedure success endpoints is challenging and has been widely discussed by many scientific societies. Experts agree that measures of outcome should be tailored to fit studied population. They have to be chosen in accordance with the class and etiology of AF, comorbidities, risk factors, and symptoms of the studied population [17, 30]. Defining strict Holter or ECG monitoring time endpoints is extremely challenging. Previous studies have shown that short-term ECG monitoring may not detect all AF episodes and therefore give inadequate information about arrhythmia presence. Various methods including intermittent short-term ECGs or prolonged monitoring have shown to be more adequate for AF detection [31, 32]. In patients after cerebrovascular event, the newest guidelines recommend to consider AF monitoring lasting even up to 30 days [33]. Therefore, a 24-h monitoring in patients after catheter ablation cannot be predictive for the procedure success outcome. In the present study, we decided to use more extensive criteria to define recurrence of the arrhythmia. In addition to the standard, recommended ECG Holter 6 and 12 months after the procedure, we decided to include in the analysis all additional ECG recordings obtained during the follow-up period which were scheduled by the managing clinicians, and what is exceptional in this study are also patients’ self-reported symptoms. In every day clinical practice, it is impossible to provide long-term monitoring devices to every AF patient; furthermore, an instant ECG is not accessible for every patient who experiences AF symptoms at the moment. We believe that if we consider symptom recurrence as an arrhythmia episode recurrence, we can improve post-procedural AF detection. For the same reasons, we decided to provide information on total success rate instead of time-specific information (i.e., 6 or 12 months). The fact that the arrhythmia was not detected in the time period does not prove that it was not present.

As for the results of the procedure, the success rate in the whole study population was relatively low, but we must remember that endpoint definitions were stricter and follow-up period was longer than in the previous studies. The majority of the studies performed in the past used risk scales like Berlin Questionnaire or Epworth Sleepiness Scale for OSA diagnosis, which are based only on OSA symptoms [34, 18]. In the present study, we base our observations solely on AHI, irrespectively of symptoms. AHI is independently associated with the disease severity and therefore corresponds better with the clinical status.

The study has several limitations; first of all, only a single night of polygraphy study was performed, which may cause to overlook some night-to-night variations. Nevertheless, to our knowledge, our study is the first study with cross-sectional design, aimed to determine the impact of OSA on sinus rhythm after AF ablation in a group of unselected patients screened with polygraphy. We were therefore able to show a real-life model of the arrhythmia impact because OSA is still largely underdiagnosed, and patients, especially asymptomatic, are rarely screened for the disease. Possibly, early screening, and treatment for OSA in AF patients, may improve strategies for selection of patients suitable for AF ablation procedure.

In conclusion, the study showed that OSA is highly prevalent in patients with AF. The presence of OSA lowers chances on successful AF ablation. The rate of arrhythmia recurrence is rising with the AHI score, which is a manifestation of the disease severity.
